# Lignin Nanoparticles with Entrapped *Thymus* spp. Essential Oils for the Control of Wood-Rot Fungi

**DOI:** 10.3390/polym15122713

**Published:** 2023-06-17

**Authors:** Florian Zikeli, Anna Maria Vettraino, Margherita Biscontri, Sara Bergamasco, Cleofe Palocci, Miha Humar, Manuela Romagnoli

**Affiliations:** 1 Department for Innovation in Biological, Agro-Food and Forest Systems (DIBAF), University of Tuscia, 01100 Viterbo, Italy; vettrain@unitus.it (A.M.V.); margherita.biscontri@unitus.it (M.B.); sara.bergamasco@unitus.it (S.B.); 2 Department of Chemistry, Sapienza University of Rome, Piazzale A. Moro 5, 00185 Rome, Italy; cleofe.palocci@uniroma1.it; 3 Research Center for Applied Sciences to the Safeguard of Environment and Cultural Heritage (CIABC), Sapienza University of Rome, P.le A. Moro 5, 00185 Rome, Italy; 4 Department of Wood Science and Technology, Biotechnical Faculty, University of Ljubljana, Jamnikarjeva 101, 1000 Ljubljana, Slovenia; miha.humar@bf.uni-lj.si

**Keywords:** wood decay, white-rot fungi, brown-rot fungi, beech, lignin nanoparticles, essential oils

## Abstract

After decades of utilization of fossil-based and environmentally hazardous compounds for wood preservation against fungal attack, there is a strong need to substitute those compounds with bio-based bioactive solutions, such as essential oils. In this work, lignin nanoparticles containing four essential oils from thyme species (*Thymus capitatus*, *Coridothymus capitatus*, *T. vulgaris*, and *T. vulgaris* Demeter) were applied as biocides in in vitro experiments to test their anti-fungal effect against two white-rot fungi (*Trametes versicolor* and *Pleurotus ostreatus*) and two brown-rot fungi (*Poria monticola* and *Gloeophyllum trabeum*). Entrapment of essential oils provided a delayed release over a time frame of 7 days from the lignin carrier matrix and resulted in lower minimum inhibitory concentrations of the essential oils against the brown-rot fungi (0.30–0.60 mg/mL), while for the white-rot fungi, identical concentrations were determined compared with free essential oils (0.05–0.30 mg/mL). Fourier Transform infrared (FTIR) spectroscopy was used to assess the fungal cell wall changes in the presence of essential oils in the growth medium. The results regarding brown-rot fungi present a promising approach for a more effective and sustainable utilization of essential oils against this class of wood-rot fungi. In the case of white-rot fungi, lignin nanoparticles, as essential oils delivery vehicles, still need optimization in their efficacy.

## 1. Introduction

The interest in wood as the construction material of the future is steadily increasing not only because of its role in CO_2_ sequestration into woody tissue but also because of the 50% reduction in a new building’s carbon footprint when concrete or steel is replaced by wood [1]. There are still obstacles for the general acceptance of wood-based buildings, which are related to the risk of degradation by wood-rot fungi, especially when considering less durable wood species such as beech, which is abundantly present in all of Europe and suitable for the bio-building sector [2,3]. Wood preservation is imperative to extend the service life of wood products. The strategies based on the use of toxic chemical wood preservation agents, such as polycyclic aromatic hydrocarbons containing creosote or chromated copper arsenate, which have a great impact on the environment, must be replaced by eco-friendly strategies [4]. The most used methods for wood preservation are still based on fossil-based organic compounds [5,6,7], non-renewable inorganic salts, or combinations of the two [8,9]. Innovative methods also comprise the application of nanomaterials, although the possible effects on human and environmental health need to be carefully assessed, as recently reviewed by Papadopoulos and Taghiyari [10]. In this context, the interest for more environmentally friendly solutions for wood preservation, such as thermal and other modification techniques [11,12,13,14], as well as impregnation with natural products, has rapidly increased [15,16,17,18]. Among natural bioactive compounds, essential oils (EOs) have gained considerable interest from researchers regarding their application as biocides [19,20,21,22,23,24,25,26,27,28,29,30,31,32,33,34,35,36,37,38]. EOs have been used since Ancient Egypt where plants were extracted using animal fats and vegetable oils, and their use increased after the invention of steam distillation at approximately 1000 AD in Arabia, which set the standard industrial method for EO extraction of today. In the first half of the 20th century, aromatherapy was founded, and early research demonstrated the therapeutic properties of EOs that, consequently, led to the treatments of various medical conditions, such as cancer, pain, stress, and infectious diseases, utilizing EOs today [39].

EOs can contain up to several hundreds of individual compounds, and through this large palette of molecules with various functional moieties and their respective chemical and physiological properties, EOs exhibit a multi-target activity in contrast to single chemical compounds [40]. Thus, these mixtures of plant secondary metabolites, such as terpenes, aldehydes, alcohols, ketones, and phenols, play an important role in growth inhibition of a wide range of human, as well as plant, pathogens. Further, broad antiviral properties of EOs were reported, where EOs stimulate immune response and, at the same time, suppress inflammation caused by viral infection. Thus, EOs were also utilized for supplemental treatments against COVID-19, causing rapid viral clearance, reduced fatigue, as well as shorter recovery times, as recently reviewed by [41]. The antibacterial activities of EOs comprise the destruction of cell membranes and eventual leakage of cell contents, damage of genetic material of microorganisms, inhibition of enzymes responsible for their metabolism, and consumption of ATP stored in their cells [42,43]. Regarding plant fungal pathogens, the mycelia growth of *Botrytis cinerea*, *Penicillium italicum*, and *P. digitatum* can be significantly reduced by EOs from oregano (*Origanum vulgare* L. ssp. *hirtum*), thyme (*Thymus vulgaris* L.), and lemon (*Citrus limon* L.) [44]. EOs from clove (*Syzygium aromaticum*), lemongrass (*Cymbopogon citratus*), mint (*Mentha* × *piperita*), and eucalyptus (*Eucalyptus globulus*) inhibit tomato wilt by *Fusarium oxysporum* f. sp. *lycopersici* 1322 [45]. EO from *Pinus rigida* wood completely inhibited the growth of the common wood mold fungi *Alternaria alternata*, *Fusarium subglutinans*, *Chaetomium globosum*, and *Aspergillus niger*, while *Eucalyptus camaldulensis* leaf EO showed inhibitory effects against *F. subglutinans* and *C. globosum* [46]. Dill seed EO inhibited mycelium growth and sclerotial germination of *Sclerotinia sclerotiorum* in vitro and suppressed *S. sclerotium* on infected oilseed rape leaves in vivo [47]. 

One of the advantages when utilizing EOs as biocides is that most of the terpenoids and phenols found in the plant EOs are relatively less toxic to humans and the environment than are synthetic chemicals [48]. Accordingly, thyme EO, as well as its principal components thymol and carvacrol, is generally recognized as safe for use in minimum-risk pesticides, and there are no significant adverse effects known to human health and the environment [49,50]. Since thyme EO and the thymol and carvacrol it contains are readily biodegradable in air, water, and soil, their utilization as biocides for wood protection is not expected to cause negative environmental effects [51]. *Thymus* spp. EOs, in general, consist of the two phenolic monoterpenes thymol and carvacrol, the aromatic terpenoid *p*-cymene, and the terpene y-terpinene, but the contents of these compounds can vary strongly depending on the respective thyme species as well as on the respective cultivation methods and locations. For Tunisian *T. capitatus* EO, carvacrol contents of 62–83% were reported [52,53], while the major component in *T. capitatus* EO from Sardinia was thymol with 29.3% [54]. The great variability of the main components is supported in a study by Miceli et al., who collected samples of *T. capitatus* at 23 different sites in Southern Apulia, Italy, where thymol contents ranged from 1 to 72%, and the carvacrol contents ranged from 7 to 74% [55].

Despite significant advances in the use of EOs against plant pathogens, only a few studies have investigated the antifungal effect of thyme EOs against specific wood-rot fungi and showed promising bioactivity against white-rot fungi, such as *Trametes versicolor* and *T. hirsuta*, and against the brown-rot *Laetiporous sulphureus* and the wet-rot *Coniopohora puteana* [25,56,57]. In a recent work, the authors studied in vitro the antifungal effects of pure thyme EOs against brown-rot (*Gloeophyllum trabeum* and *Poria monticola*) as well as white-rot fungi (*T. versicolor* and *Pleurotus ostreatus*) and proved their efficiency as biocides against wood-rot fungi [22]. Other alternatives for greener wood protection approaches are the substitution of fossil-based components in commonly used wood protection formulations with bio-based additives or, e.g., the utilization of lignin nanoparticles (LNPs) as coatings or as carriers of bioactives [4,25,58,59,60,61,62,63,64,65,66,67,68,69].

Recently, LNPs have been investigated in detail as nano- or microcarriers in the design of biocide delivery systems in agricultural or pharma applications [70,71,72,73,74,75,76,77,78,79]. Lignin qualifies as a carrier material due to its intrinsic properties as an aromatic phenolic macromolecule. It can provide protection to UV-sensitive loaded compounds and prevent the oxidation of the loadings due to its anti-oxidant properties as well as provide an unwanted fast evaporation of the volatile bioactive loadings, such as in the case of volatile essential oils [76,80]. LNPs were successfully tested as carriers and for the eventual controlled release of fungicides and plant growth regulators [81,82]. Others additionally took advantage of the UV-protective properties of lignin to prepare LNPs containing the photosensitive abscisic acid for its controlled release for plant growth stimulation [83]. The literature reports regarding the encapsulation of EOs into LNPs, however, are rather rare. Chen et al. prepared pickering emulsions of EOs containing cinnamaledhyde and eugenol stabilized by LNPs and applied them for the post-harvest protection of oranges, where fruit decay by *Penicillium italicum* was reduced by almost 50% when EOs were stabilized by LNPs [84]. Another research work utilized lignin for the encapsulation of orange EO and found an increased toxicity of orange EO against the pest insect *Spodoptera frugiperda* compared with non-encapsulated orange EO in the conducted bioassay [85].

In recent works, Zikeli et al. successfully tested the entrapment of cinnamon and thyme EOs into LNPs, and the successive delayed release of the entrapped EOs [24]. Additionally, LNPs with entrapped thyme EOs were successfully applied for controlling *Phytophtora cactorum* diseases [23]. 

The aim of this study was to apply EOs from *Thymus* spp. entrapped into LNPs as antifungal additives in the cultivation substrate of the two white-rot fungi *T. versicolor* and *P. ostreatus* and of the two brown-rot fungi *G. trabeum* and *P. monticola*, respectively. Entrapment of EOs in LNPs for their utilization in industrial wood preservation applications will reduce treatment costs significantly and are expected to have a positive effect on their efficiency as biocides due to the decreased release rate and longer duration of their biocidal effect. To our best knowledge, this is the first time that EO-containing LNPs are applied as biological control agents against wood-rot fungi. 

## 2. Materials and Methods

### 2.1. Essential Oils Containing Lignin Nanoparticles Preparation

Organsolv lignin (OSL) from beech wood was supplied by Fraunhofer CBP (Leuna, Germany). Acetone (HPLC grade) and EtOH (96%, ACS reagent grade) were purchased from Carlo Erba reagents (Cornaredo, Italy). Seamless cellulose dialysis tubing with a MWCO of 12 kDa was purchased from Sigma-Aldrich (Merck KGaA, Darmstadt, Germany).

Essential oils (EOs) from *Thymus capitatus* (TC), *Coridothymus capitatus* (CC), *T. vulgaris* (TV), and *T. vulgaris* Demeter (TVD), identical to those used in an earlier work [22], were kindly provided by Flora srl, Florence, Italy (Table 1). The plants were cultivated either on organic farms in Spain (TC and TV) or on Demeter biodynamic (TVD) or organic farms (TV) in Italy, respectively. Flora srl extracted respective essential oils by hydrodistillation using a Clevenger-type apparatus. Chemical compositions were determined by Flora srl, using a PerkinElmer Clarus 500 GC-FIDMS system, and are reported in Table 1. 

Essential oils containing lignin nanoparticles (EOLs) were prepared according to the protocol reported in Zikeli et al. [24], with small modifications. OSL (300 mg) and respective EOs (100 mg) were dissolved in 10 mL acetone and filled into dialysis bags, which were exposed to an excess of distilled water under stirring for 2.5 h at room temperature. Dialysis time was retained long enough to get rid of acetone and short enough to prevent release of the entrapped EOs. After dialysis, the samples were kept in the refrigerator for further analysis and application. Solid content of the EOL was determined by freeze-drying of aliquots of the prepared EOL dispersions. EO contents were determined with a Ultrospec 1000 photometer (Pharmacia Biotech, GE Healthcare Europe GmbH, Milano, Italy), diluting the EOL dispersions in EtOH/water (50/50 vol%) at 280 nm against external calibration by dilutions of the respective pure EO samples. EO contents were determined in triplicate.

For preparation of the samples for SEM, three drops of the EOL suspensions were adsorbed onto a glass coverslip and air-dried at 25 °C (4 h). The cover slips were then attached to aluminum stubs using carbon tape and sputter-coated with gold in a Balzers MED 010 unit (Oerlikon Balzers, Balzers, Liechtenstein), followed by SEM analysis using a JSM 6010LA electron microscope (JEOL Ltd., Tokyo, Japan).

Release experiments were conducted using 25 µL EOL samples in 225 µL liquid fungal cultivation medium (potato dextrose broth, 24 g/L, autoclaved at 121 °C for 20 min, VWR International S.r.l., Milan, Italy) in steady state in the refrigerator. Every 24 h, a sample was taken, the EO content was quantified using UV photometry as explained above, and a cumulative release of the EO was determined. Release experiments were conducted in triple determination, and the reported values represent the average of three experiments.

### 2.2. Fungal Strains

*Trametes versicolor* (ZIML057), *G. trabeum* (ZIML018), *P. ostreatus* (ZIML030), and *P. monticola* (ZIML037) from the Department of Wood Science and Technology, University of Ljubljana (Slovenia), were cultivated on Malt Extract Agar medium (MEA, 20 g/L malt extract (Oxoid, Basingstoke, UK) and 15 g/L bacteriological agar (VWR International srl, Milan, Italy)) plates by subculture of mycelia from an active 7-day culture at 24 °C for seven days. The fungal isolates derived from the fungal collection of the Biotechnical Faculty, University of Ljubljana, and are available to research institutions on demand. Origin and details of the fungal isolates are described in the respective catalogue [86].

### 2.3. Anti-Fungal Assay

In vitro antifungal activity of EOLs on mycelia growth of the selected fungi were determined using the method described by Vettraino et al. [23]. In particular, treatments were prepared within the concentration range of 0.05–1.20 mg/mL (0.05, 0.15, 0.30, 0.60, and 1.20 mg/mL). Five different concentrations of nano-encapsulated essential oils, as well as empty LNPs (LNPs solo), were dissolved into Malt Extract Agar (MEA, 20 mL) just before it was poured into the Petri dishes (9 cm) at a temperature of 45–50 °C. A 6 mm diameter circular disk of each fungal isolate, cut from the margin of the actively growing cultures on MEA using a cork borer, was inoculated in the center of each Petri dish containing the different treatments. Negative controls had only empty nanoparticles and untreated MEA. Petri dishes were sealed with polyethylene film and incubated at 25 ± 2 °C. Mycelia growth was measured every day for 7 days. Five replicates for each treatment were performed. The minimum inhibitory concentration (MIC) was determined as the lowest concentration that completely inhibited the fungal growth. 

### 2.4. FTIR Spectroscopy

FTIR spectra of *T. versicolor*, *P. ostreatus*, *P. monticola*, and *G. trabeum* grown on MEA and MEA amended with EOLs, respectively, were recorded on a Jasco FTIR-4100 FTIR spectrometer (Jasco Corporation, Easton, MD, USA). For the evaluation of fungal growth under stress conditions, the FTIR spectra of culture medium samples with EO concentrations just below the respective MICs were taken. The mycelium samples were analyzed after thorough and repeated (3 times) washing with distilled water, followed by centrifugation and freeze-drying (−50 °C, 72 h) to remove cultivation medium residues. After grinding in an agate mortar, potassium bromide (KBr) discs were prepared with a sample concentration of 2% (wt.) using a Specac Mini-Pellets Press (Specac Inc., Fort Washington, MD, USA). The spectra were acquired in the absorbance mode in the range of 4000–400 cm^−1^, with a resolution of 4 cm^−1^ against a background of pure KBr, and 64 scans were accumulated. Raw FTIR spectra were smoothed using the Means–Movement method with a convolution width of 15, baseline-corrected (zero absorbance at 822 cm^−1^, 1815 cm^−1^, 1860 cm^−1^, 2350 cm^−1^, and 3800 cm^−1^), and normalized to the absorbance maximum in the fingerprint region at 1076 cm^−1^ using Spectra Manager software (v. 2.15.01, Jasco Corporation, Easton, MD, USA). The resulting FTIR spectra were background-corrected in order to eliminate spectral information deriving from the cultivation substrate, as described in Vettraino et al. [22]. Second-derivative FTIR spectra were produced using the Savitzky–Golay algorithm (polynomial degree 3, 11 data points) and utilized for IR band area integration as well as principal component analysis (PCA) using Spectra Manager software.

### 2.5. Statistical Analysis

In order to evaluate the effect of pure nanoparticles against fungal growth, obtained data and control data were subjected to analysis of variance (ANOVA) using the Shapiro–Wilk test, and the means were compared by the Kruskal–Wallis test (*p* > 0.05) using GraphPad Prism 5.0 (GraphPad Software, San Diego, CA, USA). PCA was applied on second-derivative FTIR spectra using the PCA Model Editor of the Spectra Manager Software. Number of principal components was 3, and the calculation ranges were 3050–2750 cm^−1^ and 1835–825 cm^−1^.

## 3. Results and Discussion

### 3.1. Characterization of Lignin Nanoparticles Loaded with Essential Oils (EOL)

The detailed compositions of the utilized essential oils and their percentages are provided in Table 1. The three terpenoids—carvacrol, thymol, and *p*-cymene—can be considered as key compounds, as at least one of them is present in high concentrations in each of the four EOs. Further description of the contained substance classes is already presented in detail in Vettraino et al. [22].

The prepared EOLs had solid contents of 15.7–17.3 mg/mL, and the respective EO contents ranged from 5.0 to 7.5 mg (Table 2). The resulting drug-loading efficiencies (DLE) were between 50 and 75%, with the highest value for TVD and the lowest for CC. Drug-loading capacities (DLC) were in the same range for EOL-CC and EOL-TV, respectively, while the values were higher for EOL-TC and EOL-TVD. The results for DLE and DLC were in good accordance with those reported in an earlier work for *T. vulgaris* and *T. serpyllum*, respectively, where acidolysis lignin from beech wood was used in contrast to the technical Organosolv beech lignin utilized in this study, indicating little influence of the lignin extraction process on the EO entrapment into the lignin matrix [24].

**Figure 1:**
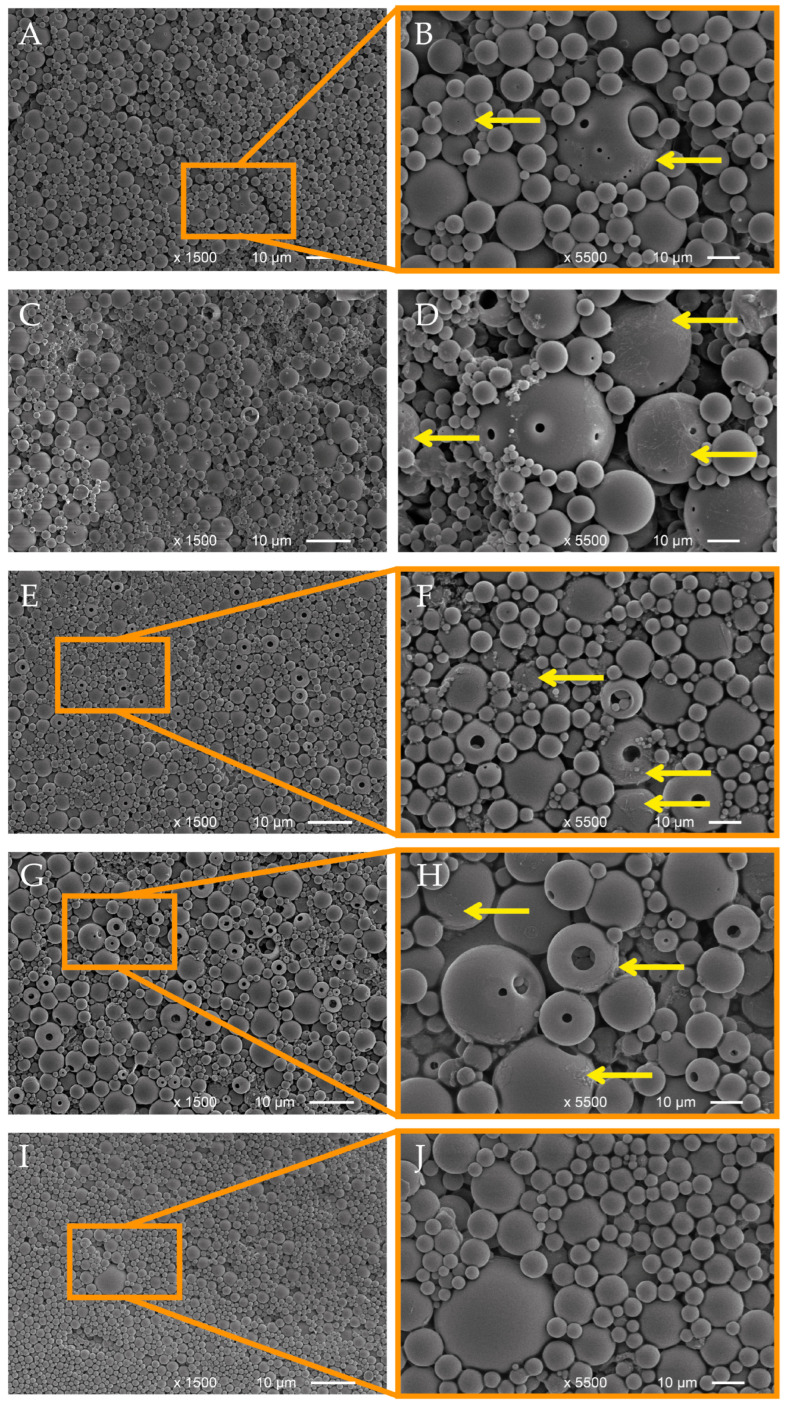
SEM images of lignin nanoparticles containing essential oils from *T. capitatus* (**A**,**B**), *T. vulgaris* (**C**,**D**), *C. capitatus* (**E**,**F**), *T. vulgaris* Demeter (**G**,**H**), and empty LNPs (**I**,**J**). Yellow arrows indicate film-like structures.

SEM analysis revealed LNPs with a high polydispersity, ranging from 100 to 200 nanometers to several micrometers (Figure 1). EOLs showed surface pores and hollow shapes, and some larger particles were observed that contained smaller particles inside them and, in higher magnifications, film-like structures were observed on the EOL surface, which were attributed to EOs incorporated into the shells of the particles (Figure 1B,D,F,H, yellow arrows). This assumption was confirmed by the absence of these film-like structures on the respective SEM photos of the empty LNPs (Figure 1J).

**Figure 2:**
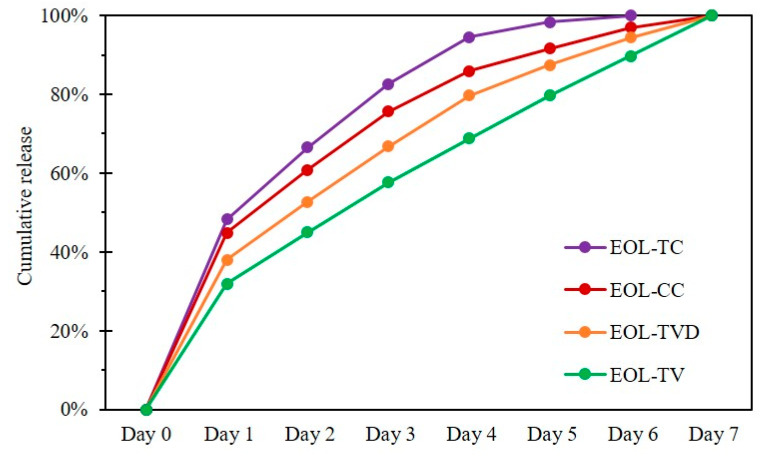
Cumulative release of essential oils from lignin nanoparticles containing essential oils from *C. capitatus* (EOL-CC), *T. capitatus* (EOL-TC), *T. vulgaris* Demeter (EOL-TVD), and *T. vulgaris* (EOL-TV).

Release experiments were designed in order to prove the retention of the bioactive compound inside its carrier matrix and its eventual gradual release over a prolonged time. The release behavior of the prepared EOLs was tested in a standard liquid cultivation medium for fungi in order to simulate fungal growth conditions and observe their effect on the release of the EOs from the lignin nanoparticles. The cumulative release was determined for 7 days, with the respective cumulative release rates reported in Figure 2. For all four EOL samples, the initial release from Day 0 to Day 1 was higher than the later daily release rates. In the SEM images, film-like structures formed from EOs were observed (Figure 1), which could be responsible for the rather large portion of EOs released during Day 1 of the release experiment, while EOs incorporated in deeper layers of the LNP matrix were assumed to be retained for a longer time. Interestingly, the release rate of EOL-TC was already higher from Day 1 onward, resulting in almost 100% release already after five days. EOL-CC also showed a high initial release rate, but the release relatively slowed down afterwards, reaching almost 100% after six days. The release rate of EOL-TVD initially was rather low, with <40% release after one day but also reaching approximately 95% after six days of the experiment. In contrast, EOL-TV showed a quite different release, with the lowest release after one day (32%) and an almost constant release rate until Day 7, indicating that even after 7 days of release, there was still EO-TV contained inside the lignin nanoparticles. Interestingly, EOL-TC and EOL-CC, which consisted of an elevated content of the single component carvacrol, showed a higher initial release rate than EOL-TV and EOL-TVD, respectively, which contained more thymol and *p*-cymene, respectively (Table 1). The different chemical structures of the compounds might interact to a different extent with the lignin carrier material, which results in a faster or slower release from the LNPs. Considering the chemical structures of the compounds contained in the EOs, the non-phenolic terpenoid *p*-cymene, whose content was higher in EOL-TVD and EOL-TV, respectively, seemed to contribute to a stronger interaction of the EOs with the lignin carrier. Compared with an earlier study [24], where full release was already achieved after 72 h, it must be stated that the release experiment method used strongly affects the outcome. While in [24], the release experiment was conducted at room temperature and under magnetic stirring, in this study, the release was assessed in a steady state and in the refrigerator to prevent the spoilage of the utilized fungal growth medium. 

### 3.2. In Vitro Inhibition Experiments

The fungal growth of *T. versicolor*, *G. trabeum*, *P. ostreatus*, and *P. monticola* was not significantly affected by the empty LNPs (Kruskal–Wallis statistics, *p* = 0.07; *p* = 0.10; *p* = 0.98; *p* = 0.56, respectively). In contrast, the tested EOs entrapped in the LNPs showed an inhibition effect against the pathogens, with MIC values ranging from 0.05 mg/mL (*P. monticola* treated with EOL-TC) up to 0.60 mg/mL (*P. ostreatus* treated with EOL-TVD and *T. versicolor* treated with EOL-TVD and EOL-CC) (Table 3). Of all the EOs investigated, EOL-TVD had the highest median MIC across all four tested strains (median 0.3 mg/mL; 0.15–0.60 mg/mL). In accordance with previous studies, a considerable variation in the in vitro inhibitory effects of the different EOs investigated was observed. To a lesser extent, the observed differences also depended on the pathogen investigated [22,87]. The entrapment of the EOs resulted in a higher inhibition effect against *G. trabeum* and *P. monticola* mycelia growth than treatments with free EOs, while no differences were observed for the treatments against *T. versicolor* and *P. ostreatus*, respectively. The ability of the two white-rot fungi *T. versicolor* and *P. ostreatus* to degrade lignin most likely caused the absence of a visible effect on the determined MIC of the entrapped EOs compared with pure EOs in contrast to the brown-rot strains. A positive effect of EOs entrapment into the LNPs also in the case of the white-rots might be achieved when extending the experimental time to a point, where all of the lignin carrier material is consumed, and the EOs are fully released. Another approach to increase efficacy against white-rot may be to decrease the ratio of lignin to EOs for the preparation of the respective EOLs so that the biocide delivery system provides relatively less substrate for the white-rot fungi. 

### 3.3. FTIR Spectroscopy

**Figure 3:**
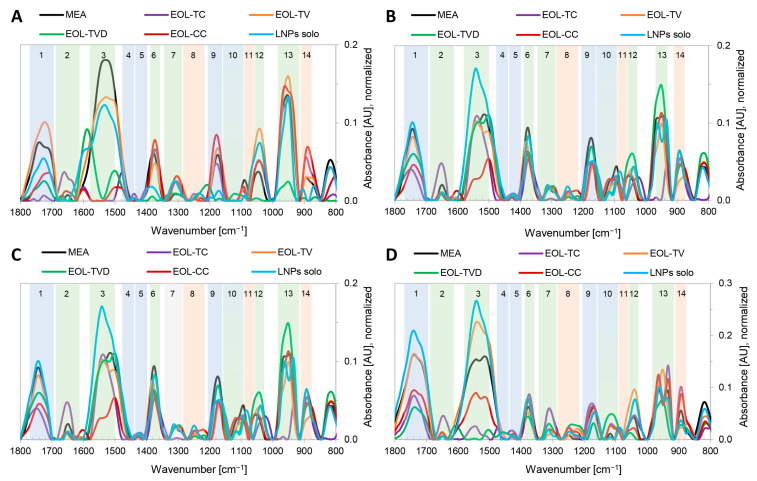
FTIR spectra, background and baseline-corrected, of *Gloeophyllum trabeum* (**A**), *Poria monticola* (**B**), *Pleurotus ostreatus* (**C**), and *Trametes versicolor* (**D**) grown on pure MEA as well as on MEA containing lignin nanoparticles with essential oils from *T. capitatus* (EOL-TC), *T. vulgaris* (EOL-TV), *T. vulgaris* Demeter (EOL-TVD) and *C. capitatus* (EOL-CC) in concentrations below the respective minimum inhibitory concentrations.

Figure 3 shows the FTIR spectra of the four investigated fungi grown under control conditions on pure MEA (MEA) and on MEA containing lignin nanoparticles with the four applied thyme essential oils entrapped. To maintain their ability to carry out specialized and life-essential functions in the cell, microorganisms are used to adapt to changes and stressful events in their environment by variation of their cell wall composition in order to keep the metabolic processes up and running, which is essential for their survival. Thus, the detection of intensity differences of IR bands specific to their cell wall compounds indicate modifications in the cell wall composition [88]. IR bands were assigned to specific compounds in the fungal cell wall—lipids (bands 1, 4, 5, and 9), polyphosphates (bands 8, 11, and 13), and chitin/carbohydrates (bands 2, 3, 6, 7, 10, 12, and 13)—based on the respective functional groups (Table A2) and according to the respective literature [89,90,91,92,93,94,95]. Regarding *G. trabeum* (Figure 3A), the largest changes were observed at the IR bands 1 (lipids), 3 (chitin), 12 (chitin), and 14 (polyphosphates). For EOL-TV, the absorbance at IR band 1 increased, and it decreased for all other MEA formulations, being almost eliminated in the case of EOL-CC. The absorbance of IR band 14 was strongly affected by the applied EOLs compared with pure MEA. This could mean that the structure of the fungal cell wall phospholipid layer was significantly influenced by the presence of the respective EOLs in the culture medium. The intensity of the chitin-related IR band 3 was strongly reduced for EOL-TC, EOL-CC, and EOL-TVD, while the absorbance loss was lower for EOL-TV and the empty LNPs. Further, increases in absorbance were registered for IR band 12 for all formulations except EOL-TVD, indicating an increased chitin synthesis as a possible defense mechanism of *G. trabeum* against stress caused by the EOs entrapped in the LNPs. 

In the overlay of the FTIR spectra of *P. monticola* grown on the different substrates in Figure 1B, the respective spectrum of the fungus grown on MEA containing EOL-CC is missing because there was 100% inhibition, even in the lowest used concentration of EO-CC. The respective background and baseline-corrected FTIR spectrum is, therefore, considered to be a straight horizontal line of zero absorbance (Figure 3B, EOL-CC) since there was no growth and, therefore, no mycelium of *P. monticola* in the cultivation substrate. The FTIR spectra of *P. monticola* grown on modified MEA showed absorbance decreases at IR band 1 (lipids) for all four EOs. Further, changes in the chitin-related IR band 3 as well as the IR band 8 related to polyphosphates were observed. The absorbance of IR band 3 became slightly lower for EOL-TC and slightly higher for EOL-TV and the empty LNPs, respectively. The IR band 8 showed higher absorbance for EOL-TV, EOL-TVD, as well as the empty LNPs. Considering simultaneous changes in the lipid IR band 1 and the poylphosphate IR band 8, the EOs apparently had an effect on the phospholipid layer of the cell wall of *P. monticola*.

The white-rot fungus *P. ostreatus* showed stronger absorbance differences at the IR bands 1, 3, 6, and 9 (Figure 3C). Similar to those of *G. trabeum* and *P. monticola*, an absorbance decrease in IR band 1 (lipids) was registered for EOL-CC, EOL-TC, and EOL-TVD, respectively, while for the empty LNPs and EOL-TV, the absorbance remained in the range of the control sample. There were absorbance decreases in another lipid IR band (9) for all samples except EOL-TVD when compared with the control sample. Further, the chitin-related IR bands 3 and 6 showed differences: the formulation with EOL-CC had a strong absorbance decrease, while the other EOs remained at the intensity of the control sample, and the pure LNPs caused an absorbance increase. 

Similar to the other three fungal strains, *T. versicolor* showed IR absorbance changes at IR band 1 and 3 compared with the control experiment on pure MEA (Figure 3D). Additionally, changes were detected at the IR bands 10, 12, and 14. At IR band 1, an absorbance decrease was registered for the MEA formulations containing EOL-CC, EOL-TC, and EOL-TVD, while an increase was observed for MEA containing the empty LNPs. Regarding the chitin-related IR band 3, the EOLs from TC, CC, and TVD caused a strong absorbance decrease, while EOL-TV and the empty LNPs led to a higher absorbance than for pure MEA. The polyphosphates IR band 14 was reduced for EOL-TV, EOL-TVD, and the empty LNPs, while the absorbance of this band was larger than in pure MEA when adding EOL-CC and EOL-TC, respectively. Simultaneous changes in the lipids as well as the polyphosphate-related IR band indicated a disturbance in the phospholipid biosynthesis of *T. versicolor* caused by the different formulations. Additionally, the changed absorbance in the chitin-related bands indicated a modified chitin biosynthesis as a stress response to the added EOs.

In all in vitro inhibition experiments, changes in band 1 (lipids) were observed, namely a decrease in IR absorbance when the EOLs were used. This is in contrast to the earlier work of the authors, in which a respective IR absorbance increase in the lipid band was observed in the presence of pure EOs [22], which is a known fungal response to stressful events in order to protect the cell membrane and the proteins contained therein [95]. This indicates that the effect on the fungi was different in the presented work when the EOs were applied entrapped inside the LNPs. Lignin is known to non-productively adsorb polysaccharide hydrolases by hydrophobic-, electrostatic-, or hydrogen-bonding interactions when they are used for enzymatic digestion of biomass [96]. It could, therefore, be speculated, that the presence of LNPs could somehow inhibit or deactivate the conventional fungal response and the respective enzymes responsible for an increased lipid synthesis to protect their cell wall against stress caused by EOs.

**Figure 4:**
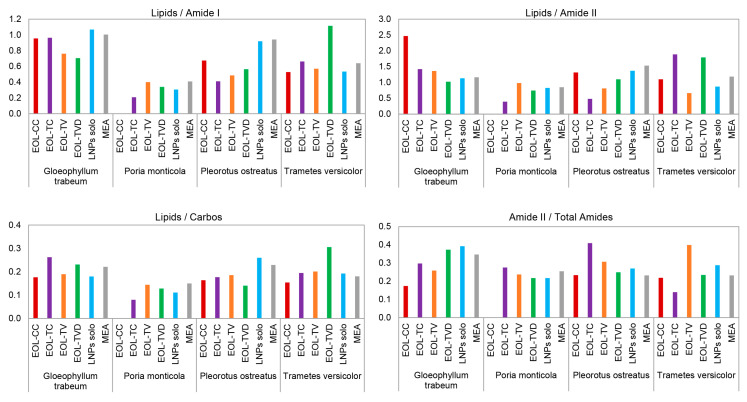
IR band area ratios of the second derivatives of the background-corrected FTIR spectra of the four fungi grown on pure MEA (MEA) and on MEA containing empty lignin nanoparticles (LNPs solo) as well as LNPs containing the essential oils of the four thyme species (EOL-CC, EOL-TC, EOL-TV, EOL-TVD).

When analyzing the band area ratios of the respective second derivatives of the FTIR spectra (Figure 4), it was observed that in the spectra of the fungi grown on MEA containing the empty LNPs (LNPs solo), the ratio of Lipids/Amide I decreased just a little (*P. monticola*, *P. ostreatus*, *T. versicolor*) or even increased (*G. trabeum*). On the basis of Figure 1, it was already observed that the lipid-related IR band 1 had a similar absorbance intensity for MEA containing the empty LNPs (LNPs solo) compared with the control samples for all four fungi. Therefore, this can be confirmed by the FTIR data of the second derivatives illustrated in Figure 4, where the lipid band at 3000–2800 cm^−1^, which lies outside the fingerprint region of the respective FTIR spectra, was also taken into account. In contrast to the empty LNPs, in the FTIR spectra of the four fungi grown on MEA containing the EOLs, the ratio of Lipids/Amide I decreased in almost all the cases compared with the respective control samples. In just two cases (*T. versicolor* vs. EOL-TVD and *T. versicolor* vs. EOL-TC), this ratio increased compared with the control experiment. In three other cases, the ratio of Lipids/Amide I remained at the level of the control sample (*G. trabeum* vs. EOL-CC, *G. trabeum* vs. EOL-TC, and *P. monticola* vs. EOL-TV). The general decrease in the ratio of Lipids/Amide I in the EOL-containing MEA formulations compared with pure MEA was already indicated by the observations of the respective FTIR spectra in Figure 2, where the absorbance of the lipid-related IR band 1 decreased in most cases. When analyzing the IR band ratio of Lipids/Amide II, the general pattern was similar to the ratio of Lipids/Amide I for *P. monticola* and *P. ostreatus*, respectively. In the case of *G. trabeum*, the ratio of Lipids/Amide II increased for MEA containing EOL-CC, EOL-TC, and EOL-TV, which correlated with the strong absorbance decrease in IR band 3 observed in Figure 3A. In the case of *T. versicolor*, a strong increase in the ratio of Lipids/Amide II was registered for MEA containing EOL-TC and EOL-TVD, respectively. 

When considering the MIC values calculated for EOL-TC and EOL-TVD against *T. versicolor* (Table 3), it is evident that an increase in the IR band ratio of Lipids/Amide II, which indicated a stress reaction of the fungus, was not always in correlation with a growth inhibition of the fungus by the applied EOLs. Although the IR band ratio of Lipids/Amide II was highest for EOL-TVD, the MIC values were lower for EOL-TC and EOL-TV, respectively, meaning that EOL-TVD was less effective against *T. versicolor*. On the contrary, the high Lipids/Amide II ratio of EOL-TC against *T. versicolor* correlated with the low MIC determined for this case (Table 3). When observing the Lipids/Carbos IR band ratio of the fungi grown on MEA containing the empty LNPs, it became evident that for the brown-rot fungi *G. trabeum* and *P. monticola*, the ratio decreased compared with that of pure MEA, while it increased for the white-rot fungi *P. ostreatus* and *T. versicolor*. Their ability to degrade lignin could be a reason for the different response to the LNPs in the MEA terrain observed for the white-rot strains. The processing of lignin can provide additional acetyl-CoA and succinyl-CoA that, in turn, feed their central metabolism for lipid biosynthesis, for example, as an eventual stress response leading to a higher Lipids/Carbos IR band ratio [97,98,99,100]. On the contrary, it could be speculated that the presence of the LNPs might disturb lipid synthesis in brown-rot fungal cell walls via the inhibition of enzymes responsible for the construction of the phospholipid layer in the cell wall, as pointed out above. While the ratios of Lipids/Carbos for EOL-CC and EOL-TVD were in the range of the empty LNPs and lower than in the control MEA for *G. trabeum*, it was higher than in the control MEA when the cultivation medium contained EOL-TC or EOL-TV, respectively. This could have been caused by a relatively greater stress imposed by EOL-TC and EOL-TV on *G. trabeum*, triggering a lipid accumulation stress response, to a certain extent, which was not disturbed by the amount of LNPs present in the cultivation medium. While the IR band ratio of Amide I/Total Amides showed only slight variations between pure and EOL-containing MEA (Table A1), respectively, the ratio of Amide II/Total Amides varied much more, which means that the EOs affected the Amide II band to a greater extent, which corresponds to IR band 3 in Figure 2. Figure 4 shows that the ratio of Amide II/Total Amides decreased for EOL-CC, EOL-TC, and EOL-TV, while it increased for EOL-TVD and the empty LNPs compared with pure MEA in the case of *G. trabeum*. *P. monticola*, instead, showed low variations for both the IR band ratios of Amide I/Total Amides and Amide II/Total Amides. Interestingly, *P. ostreatus* had the largest variation in the case of MEA with added EOL-TC, but the MIC results were equal to those for EOL-CC and EOL-TV, respectively, which showed a much lower change in the Amide II/Total Amides ratio. In the case of *T. versicolor*, the strongest variations in this IR band ratio were registered for EOL-TV and EOL-TC, which correlated with the MIC results that were lowest for those two substrate formulations. Interestingly, the ratio was almost the same for pure MEA and MEA containing EOL-CC, but the respective MIC for EOL-CC was comparable to MEA containing EOL-TV, where the IR band ratio of Amide II/Total Amides was the largest. As mentioned above, the changes in the IR band ratios might not always reflect the respectively determined MICs, as changes in the cell wall structure induced by the fungi might not affect its growth but instead should allow the fungus to cope with the stress induced by the present EOLs.

**Figure 5:**
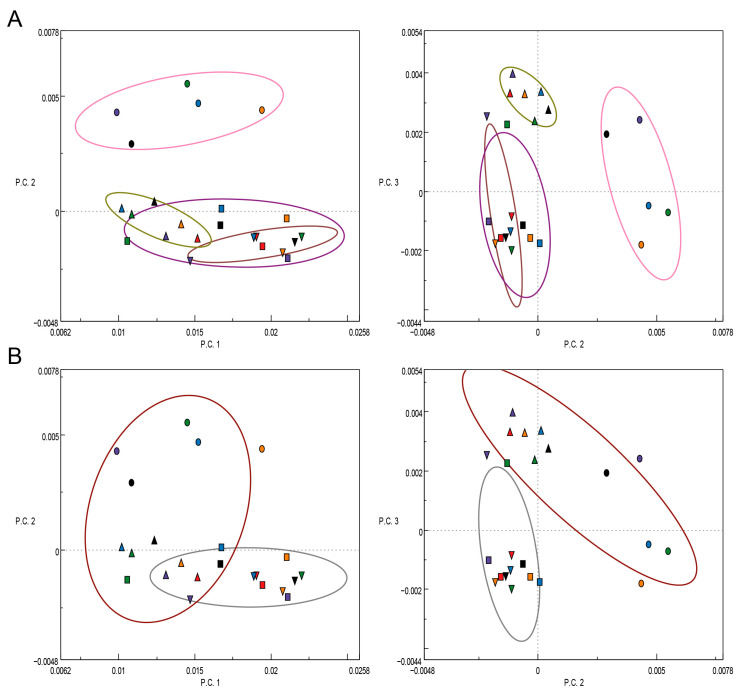
PCA scatter plots of the first three principal components (P.C.1 vs. P.C.2, left, and P.C.2 vs. P.C.3, right) of the second derivatives of the background and baseline-corrected FTIR spectra of *T. versicolor* (squares), *G. trabeum* (upward triangles), *P. monticola* (circles), *P. ostreatus* (downward triangles) grown on pure MEA (Control, black) as well as on MEA with lignin nanoparticles containing essential oils from *T. capitatus* (EOL-TC, purple), *C. capitatus* (EOL-CC, red), *T. vulgaris* (EOL-TV, orange), and *T. vulgaris* Demeter (EOL-TVD, green). (**A**) Groupings according to the fungal species. (**B**) Groupings according to white-rot (grey circle) and brown-rot (brown circle) fungi.

The results of the PCA analysis of the second derivatives of the respective FTIR spectra are illustrated in a scatter plot, where the single experiments were grouped according to the different fungal strains (Figure 5). This is in contrast to an earlier work of the authors, where the pure EOs were utilized in their free form as additives of the cultivation substrate, and the single experiments were grouped according to the different EOs applied [22]. Interestingly, the single experiments of the two fungi *T. versicolor* (Figure 5, squares) and *P. ostreatus* (Figure 5, downward triangles), which are both white-rot fungi, strongly overlap in the PCA scatter plots (Figure 5B, grey circle). Their ability to degrade lignin and eventually even deactivate phenolic compounds in the EOs, such as thymol and carvacrol, via peroxidases as pointed out by Pánek et al. [56], apparently caused these two fungi to react in a similar way to the stress caused by the present EOLs. When considering the PCA scatter plot of the first two PCs (Figure 5, left), the fungus *P. monticola* (circles) grouped up in distance to the other three fungi, similar as *G. trabeum* (upward triangles), for which the grouping is clearer in the PCA scatter plot of P.C.2 and P.C.3, respectively (Figure 5A, right). A concordance of the results of the white-rot fungi was also observed in the in vitro inhibition experiments regarding the respective MIC values, as mentioned above, confirming the findings of FTIR spectroscopic investigations.

## 4. Conclusions

EOL from thyme spp. proved to be efficient as a biocide delivery system for the control of wood-rot fungi, such as *T. versicolor*, *P. ostreatus*, *G. trabeum*, and *P. monticola* in in vitro experiments. The EOL dispersions showed a delayed release of the entrapped EOs when applied in a liquid fungal cultivation medium. FTIR spectroscopy once again proved to be a useful tool to estimate the fungal cell wall changes caused by the presence of the EOs in the cultivation medium. For each one of the four wood-rot fungi, an EOL sample with satisfying efficacy was identified. EOL dispersions are now ready to be tested as wood coatings against wood-rot fungi, and the respective in vitro experiments using coated wood specimens will be conducted next. EOL dispersions from thyme showed a promising potential approach for a wide range of wood protection applications where wooden structures in indoor as well as outdoor conditions encounter humidity and the danger of an attack by wood-rot fungi.

## Figures and Tables

**Table 1:** Main chemical compounds (relative abundance) contained in the essential oils of *Thymus capitatus* (EO TC), *Coridothymus capitatus* (EO CC), *T. vulgaris* (EO TV), and *T. vulgaris* Demeter (EO TVD).

EO TC		EO CC		EO TV		EO TVD	
Component	% Peak Area	Component	% Peak Area	Component	% Peak Area	Component	% Peak Area
carvacrol	68.6	carvacrol	76.9	thymol	47.9	*p*-cymene	26.7
*p*-cymene	7.7	α-bisabolene	3.7	*p*-cymene	15.8	thymol	20.7
γ-terpinene	6.8	caryophyllene oxide	3.3	γ-terpinene	10.0	limonene	5.6
β-caryophyllene	2.6	β-bisabolene	3.2	carvacrol	4.4	α-terpinolene	5.0
β-myrcene	1.8	β-caryophyllene	2.8	linalool	4.1	carvacrol	3.8
linalool	1.5	carvacrol acetate	1.4	β-caryophyllene	2.1	β-caryophyllene	2.9
α-thujene	1.2	L-terpinen-4 ol	0.6	β-myrcene	2.0	camphene	2.3
α-terpinene	1.1	eugenol	0.3	borneol	1.3	α-pinene	2.1
α-pinene	0.9	borneol	0.3	α-terpinene	1.3	borneol	2.1
terpinene 4-ol	0.7	δ-cadinene	0.2	α-thujene	1.2	linalool	2.0
thymol	0.6	cedrenol	0.7	camphene	1.1	β-pinene	1.9

**Table 2:** Solids content, essential oil (EO) content, drug-loading efficiency (DLE), and drug-loading capacity (DLC) of empty lignin nanoparticles (LNP solo) as well as LNPs with entrapped EOs from *C. capitatus* (EOL-CC), *T. capitatus* (EOL-TC), *T. vulgaris* (EOL-TV), and *T. vulgaris* Demeter (EOL-TVD).

	Solids (mg/mL)	EO (mg/mL)	DLE (%)	DLC (%)
LNPs solo	16.7	-	-	-
EOL-CC	15.7	5.0	50	32
EOL-TC	16.7	7.1	71	43
EOL-TV	17.3	5.6	56	32
EOL-TVD	15.7	7.5	75	48

**Table 3:** Minimal inhibitory concentration (MIC) lignin nanoparticles containing the essential oils from *C. capitatus* (EOL-CC), *T. capitatus* (EOL-TC), *T. vulgaris* (EOL-TV), and *T. vulgaris* Demeter (EOL-TVD) against the four wood-rot fungi *T. versicolor*, *G. trabeum*, *P. ostreatus*, and *P. monticola*. * The table also includes data obtained in a previous study focused on the antifungal activity of the EOs against the same wood-rot fungi [22].

Fungal Strain	EOL	MIC	* MIC EOs Solo
		(mg/mL)	(mg/mL)
*T. versicolor*	EOL-CC	0.60	0.60
	EOL-TC	0.30	0.30
	EOL-TV	0.30	0.30
	EOL-TVD	0.60	0.60
*P. ostreatus*	EOL-CC	0.30	0.30
	EOL-TC	0.30	0.30
	EOL-TV	0.30	0.30
	EOL-TVD	0.60	0.60
*G. trabeum*	EOL-CC	0.30	0.60
	EOL-TC	0.30	0.60
	EOL-TV	0.15	0.30
	EOL-TVD	0.30	0.60
*P. monticola*	EOL-CC	0.15	0.30
	EOL-TC	0.05	0.05
	EOL-TV	0.15	0.30
	EOL-TVD	0.15	0.30

## Data Availability

The data presented in this study are available on request from the corresponding author.

## Appendix A

**Table A1:** IR band area ratios of the second derivatives of the FTIR spectra of the fungi grown on pure MEA and MEA containing empty LNPs as well as LNPs loaded with EOs of the four thyme species (EOL-CC, EOL-TC, EOL-TV, EOL-TVD). Spectral regions used for band area integration: Lipids, 3000–2800 cm^−1^; Amide I, 1705–1575 cm^−1^; Amide II, 1575–1480 cm^−1^; Amide III, 1350–1240 cm^−1^; Carbohydrates, 1200–900 cm^−1^.

Fungus	Substrate	Lipids/Amide I	Lipids/Amide II	Amide I/Total Amides	Amide II/Total Amides	Lipids/Carbos
*Gloeophyllum trabeum*	EOL-CC	0.955	2.465	0.448	0.174	0.177
	EOL-TC	0.963	1.422	0.439	0.297	0.263
	EOL-TV	0.762	1.363	0.463	0.259	0.190
	EOL-TVD	0.706	1.021	0.540	0.373	0.231
	LNPs solo	1.068	1.131	0.416	0.393	0.180
	MEA pure	1.004	1.163	0.401	0.346	0.221
*Poria monticola*	EOL-CC	-	-	-	-	-
	EOL-TC	0.210	0.394	0.517	0.275	0.080
	EOL-TV	0.401	0.980	0.580	0.237	0.144
	EOL-TVD	0.340	0.745	0.476	0.217	0.128
	LNPs solo	0.306	0.822	0.584	0.217	0.111
	MEA pure	0.409	0.849	0.529	0.255	0.150
*Pleorotus ostreatus*	EOL-CC	0.675	1.314	0.454	0.233	0.164
	EOL-TC	0.412	0.483	0.479	0.409	0.177
	EOL-TV	0.487	0.812	0.512	0.307	0.186
	EOL-TVD	0.565	1.101	0.485	0.249	0.141
	LNPs solo	0.920	1.367	0.401	0.270	0.260
	MEA pure	0.941	1.532	0.378	0.232	0.229
*Trametes versicolor*	EOL-CC	0.528	1.101	0.455	0.219	0.154
	EOL-TC	0.663	1.888	0.399	0.140	0.195
	EOL-TV	0.571	0.663	0.463	0.399	0.201
	EOL-TVD	1.115	1.790	0.377	0.234	0.305
	LNPs solo	0.533	0.867	0.467	0.288	0.192
	MEA pure	0.640	1.182	0.428	0.232	0.180

**Table A2:** Main IR absorption bands and signal assignments of chemical structures derived from the respective biopolymers [89,90,91,92,93,94,95].

Band Number	Wavenumber (cm^−1^)	Signal Assignment	Biopolymer Contribution
-	3500–3200	O-H stretching	carbohydrates
-	3275	N-H stretching	chitin/chitosan
-	3105	N-H stretching	chitin/chitosan
-	2955	=C-H stretching	lipids
-	2925	-C-H (CH_3_) stretching	lipids
-	2855	-C-H (CH_2_) stretching	lipids
1	1745	-C=O stretching in esters	lipids
2	1680–1630	-C=O stretching, Amide I	proteins, chitin
3	1560–1530	C-N-H deformation, Amide II	proteins, chitin
4	1465	-C-H (CH_2_, CH_3_) bending	lipids
5	1402	C=O pf COO^-^ groups	lipids
6	1377	-C-H (CH_3_) bending	chitin
7	1320	Amide III	proteins, chitin
8	1265	P=O stretching	polyphosphates, phospholipids
9	1180	C-O-C stretching in esters	lipids
10	1150	C-O and C-O-C stretching	carbohydrates
11	1075	PO_2_ symmetric stretching	polyphosphates, phospholipids
12	1043	C-O stretching	carbohydrates
13	930	glycosidic linkages	carbohydrates
14	894	P-O-P stretching	polyphosphates
